# Effect of MHC Linked 7-Gene Signature on Delayed Hepatocellular Carcinoma Recurrence

**DOI:** 10.3390/jpm11111129

**Published:** 2021-11-02

**Authors:** Fomaz Tariq, Walizeb Khan, Washaakh Ahmad, Syeda Kiran Riaz, Mahvish Khan, Subuhi Sherwani, Shafiul Haque, Muhammad Faraz Arshad Malik, Muhammad Jahangir Iftikhar, Saif Khan, Farhan Haq

**Affiliations:** 1 Department of Biosciences, COMSATS University, Islamabad 44000, Pakistan; fomazabbasi17@gmail.com (F.T.); wjzebkh@yahoo.com (W.K.); wishiahmad@gmail.com (W.A.); syedakiranriaz@gmail.com (S.K.R.); famalik@comsats.edu.pk (M.F.A.M.); 2 Department of Molecular Biology, Shaheed Zulfiqar Ali Bhutto Medical University, Islamabad 44000, Pakistan; 3 College of Medicine, Texas A&M University, College Station, TX 77840-77845, USA; 4 Department of Biology, College of Science, Ha’il University, Ha’il 55211, Saudi Arabia; mahvishkhan02@gmail.com (M.K.); susherwani@gmail.com (S.S.); 5 Research and Scientific Studies Unit, College of Nursing and Allied Health Sciences, Jazan University, Jazan 45142, Saudi Arabia; shafiul.haque@hotmail.com; 6 Faculty of Medicine, Görükle Campus, Bursa Uludağ University, Bursa 16059, Turkey; 7 Department of Cardiology, University Hospital of Wales, Wales CF14 4XW, UK; j2j_87@hotmail.com; 8 Department of Basic Dental and Medical Sciences, College of Dentistry, Ha’il University, Ha’il 55211, Saudi Arabia

**Keywords:** MHC, HCC, gene signature, HLAs, immune-modulators

## Abstract

Dysregulated immune response significantly affects hepatocellular carcinoma’s (HCC) prognosis. Human Leukocyte Antigens are key in devising immune responses against HCC. Here, we investigated how HLAs modulate HCC development at the transcriptomic level. RNA-seq data of 576 patients from two independent cohorts was retrieved. The clinicopathological relevance of all HLA genes was investigated using Fisher-Exact, correlation, and Kaplan–Meier and cox regression survival tests. Clustering of ~800 immune-related genes against HLAs was completed using a ward-agglomerative method. Networks were generated using 40 HLA associated unique genes and hub genes were investigated. HLAs including HLA-DMA, HLA-DMB, HLA-DOA and HLA-DRB6 were associated with delayed recurrence in both discovery (204 HCC cases) and validation (372 HCC cases) cohorts. Clustering analyses revealed 40 genes associated with these four HLAs in both cohorts. A set of seven genes (NCF4, TYROBP, LCP2, ZAP70, PTPRC, FYN and WAS) was found co-expressed at gene–gene interaction level in both cohorts. Furthermore, survival analysis revealed seven HLA-linked genes as predictors of delayed recurrence. Multivariate analysis also predicted that mean expression of 7-gene is an independent predictor of delayed recurrence in both cohorts. We conclude that the expression of 7-gene signature may lead to improved patient prognosis. Further studies are required for consideration in clinical practice.

## 1. Introduction

HCC is a common heterogeneous malignancy of the liver with a high mortality rate. It is second most common cause of mortality in males and overall third most common worldwide [1,2]. Despite continuously improving therapeutic approaches, the overall survival rate of HCC patients is still very low. Studies revealed that 40–80% of HCC patients will have recurrence and metastasis within 5 years after receiving treatment [1]. Therefore, a new and distinct approach is required for early-stage diagnosis and improving survival of HCC patients.

Major Histocompatibility Complex (MHC) assists the immune system in “host defense mechanism”, which comprises of genes that encode proteins present on the cell surface, also known as Human Leukocyte Antigens (HLAs) [3]. MHCs can be divided into three classes: class I, II and III. The class I region contains genes including three classical (*HLA-A*, *HLA-B* and *HLA-C*), three non-classical (*HLA-E*, *HLA-F* and *HLA-G*) and twelve non-coding genes or pseudogenes [4]. Recent studies demonstrated the association of HLA expression with the prognosis of cancer, suggesting new immunotherapeutic options. However, contradictory results are observed in HLA expression-based prognosis prediction. For instance, overexpression of *HLA-G*, an MHC class I gene, was found in high grade tumors and suggested as a new checkpoint in different cancers [5,6]. The expression of *HLA-B* and *HLA-E* showed linkage with cancer cell migration, metastasis and poor overall survival in pancreatic and endometrial cancers [7,8]. On the contrary, MHC class I genes were also linked with better prognosis in breast and gastrointestinal tumors, suggesting tissue specific roles of HLAs [9,10]. Similarly, overexpression of MHC class II genes including *HLA-DP*, *HLA-DQ* and *HLA-DR* showed association with better prognosis in lung cancer [11]. Additionally, HLA-DR expression was also suggested to improve the prognosis of metastatic breast cancer patients [12].

In HCC, MHC class II molecules are most commonly expressed [13]. Previous studies have also shown that HLAs may play a role in HCC prognosis [14,15,16]. However, due to contradictory results on prognosis prediction, no HLA based immunotherapies are suggested in clinical practice. Additionally, limited information is available regarding the linkage of major HCC associated pathways with HLA expression. Therefore, a comprehensive and integrated study is required to ascertain the prognostic role of all HLA genes in HCC as well as their linkage with genetic pathways associated with HCC.

Thus, the current study is devised to investigate the association of HLA genes with the prognosis and survival of HCC patients. For that purpose, high throughput whole transcriptomic data of 372 and 204 HCC patients along with complete clinical information was retrieved for the analysis. Initially, the clinicopathological relevance of 23 HLA genes from all classes was established. Next, the prognostic role of immune response genes linked with HLA expression was also evaluated. The current study has immense clinical implications as we classified HCC patients into different prognostic groups based on the expression of HLAs and HLA-associated immune response genes.

## 2. Materials and Methods

### 2.1. RNA Sequencing Data of 204 HCC Patients (Discovery Cohort)

**Figure 1:**
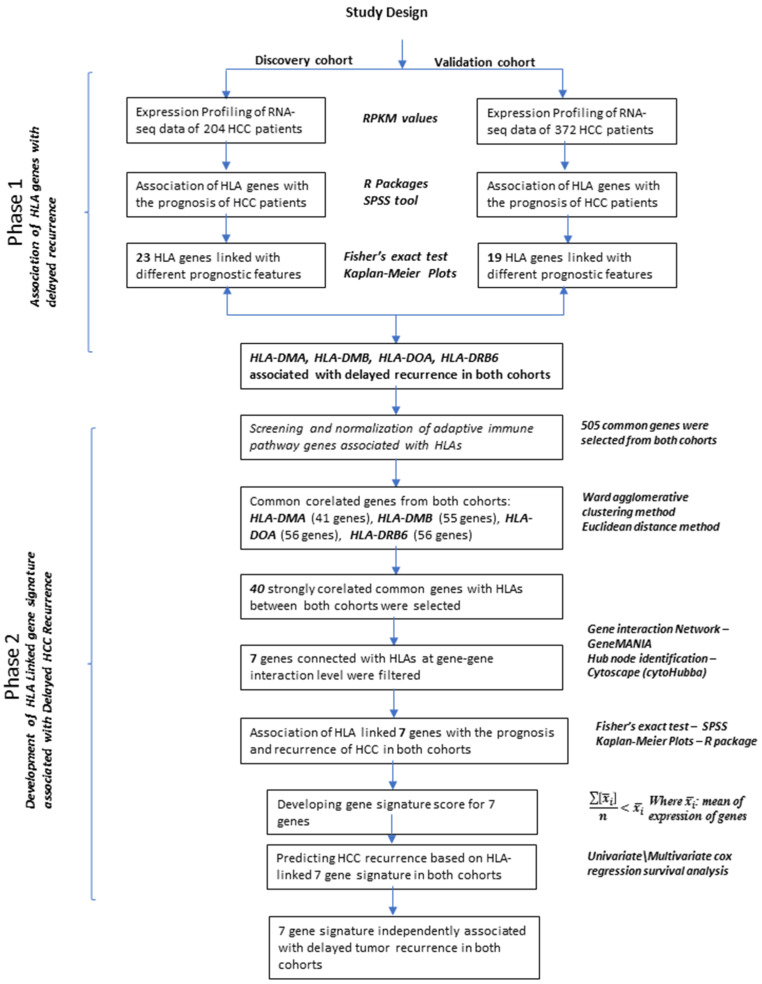
A detailed workflow of the study.

Overall study design is presented in Figure 1. The tissues samples of 204 HCCs patients diagnosed between 2005 to 2013 were obtained from the Bio-Resource Center, Korea Biobank Network. All procedures performed in studies involving human participants were in accordance with the ethical standards of the institutional and national research committee and with the 1964 Helsinki declaration and its later amendments or comparable ethical standards ((IRB # 2012–0389, ASAN Medical Center, Korea), (COMSATS University Islamabad (CIIT/Bio/ERB/16/21)). Library preparation and RNA quality assessment is described in previous studies [17,18]. Quality check was performed using FASTQC on raw FASTQ files. Alignment was done using STAR RNA-Seq Aligner v2.x7.2 [19]. Duplicates were marked using a Picard tool (https://broadinstitute.github.io/picard/ last accessed on 30 March 2020). After post-alignment clean-up, all the mapped sequences were merged into a Binary Aligned/Mapped (BAM) file. Gene expression analysis was performed using HTSEQ-count (https://htseq.readthedocs.io/en/master/ last accessed on 30 March 2020). The numbers of aligned reads that overlap each gene in the annotations were counted. Lastly, Reads per Kilobase Million (RPKM) values were generated for the analysis. To calculate z-score, = mean expression in reference sample was subtracted from each expression in the tumor sample and the result was divided by the standard deviation of expression in the reference sample [20]. Clinical information included age, sex, HBV, HCV, tumor size, Edmonson most grade, alpha-fetoprotein level, microvascular invasion, cirrhosis, fibrosis, overall survival and disease free survival (Table S1).

### 2.2. RNA Sequencing Data of 372 HCC Patients (Validation Cohort)

RPKM and z-score values of 372 HCC patients were downloaded from The Cancer Genome Atlas (TCGA) database (https://portal.gdc.cancer.gov/ last accessed on 30 March 2020) [21]. Complete clinical information of these samples was downloaded from the cBioPortal database (http://www.cbioportal.org/public-portal/, last accessed on 30 March 2020). Clinical information includes sex, HBV, HCV, tumor size, M stage, T stage, N stage, vascular invasion, cirrhosis, fibrosis, overall survival and disease free survival (Table S2).

### 2.3. Statistical Analysis

SPSS 21.0 software (IBM, Armonk, NY, USA) and R version 3.5.1 were used for all statistical analyses. Multiple statistical tests were performed including Fisher Test, Chi square Test, Kaplan–Meier survival and univariate and multivariate cox regression survival analysis. *p*  <  0.05 was considered as statistically significant.

### 2.4. Clustering and Correlation Analysis

For clustering analysis, more than ~800 immune pathway and major HCC-related pathway genes were extracted through extensive text mining and database search. Correlation and clustering analysis of those genes against HLAs were performed. Spearman test was performed for correlation analysis. Hierarchical clustering was performed using Ward’s agglomerative method [22]. HLAs in samples were indicated by a colored bar: red for upregulation and yellow for downregulation. Rows indicated clustering of genes (Figure S1).

### 2.5. Network Analysis of Candidate Co-Expressed Genes 

The gene interactions among HLA genes and immune pathway genes were retrieved using GeneMania based parameters including physical contacts, co-expression, genetic interactivity, shared protein domains and co-localization. The hub was obtained using the CytoHubba [23]. Frequently occurring hub nodes in the gene interaction network with respect to mcc centrality measure, betweenness, bottleneck and degree measure were selected for the analysis.

### 2.6. Gene Signature Score Calculation

Lastly, a signature score was calculated for the HLA-linked immune response genes which showed association with HCC prognosis. The following equation was used to calculate gene score:

$$\frac{\sum \left[{\bar{x}}_{i}\right]}{n}<\bar{x_{i}}\;\geq \;1$$

where $\;\bar{x_{i}}$: mean of expression of genes. Briefly, mean expression of all genes was calculated separately for each sample. Then mean of mean was calculated across the samples. Lastly, if the mean expression in each sample was greater than the overall mean, we gave it a score of 1. If the mean expression in each sample was less than the overall mean, we gave it a score of 0.

After generating the score for significant genes using Kaplan Meier plots and univariate, multivariate cox regression methods were applied on validation and discovery cohorts for analyzing the association of gene signature with disease free survival (DFS) and overall survival (OS). Associations with *p*-values < 0.05 were considered significant.

## 3. Results

### 3.1. Clinical Analysis of MHC Genes (Discovery Cohort)

Initially, mRNA expression of all HLA genes was screened from both discovery and validation for the analysis. Tables S1 and S2 consist of clinical characteristics of HCC patients for discovery and validation cohorts, respectively. In the discovery cohort, high expression of MHC Class I genes showed significant association with different clinical features including sex (*HLA-G p* = 0.003, *HLA-L p* = 0.018), non-HBV (*HLA-E p* = 0.004), Edmonson grade (*HLA-C p* = 0.029, *HLA-E p* = 0.029), tumor size (*HLA-L p* = 0.047) and cirrhosis (*HLA-F*, *p* = 0.034). According to survival analysis results, HLA-E (*p* = 0.041) showed association with better disease-free survival while *HLA-G* (*p* < 0.05) was associated with better disease free as well as overall survival of HCC patients (Table S3). MHC class II genes showed significant associations with multiple clinical features including age (*HLA-DPA1 p* = 0.035, *HLA-DMB p* = 0.001, *HLA-DPB1 p* = 0.017, *HLA-DQA2 p* = 0.017, *HLA-DQB2 p* = 0.000, *HLA-DRA p* = 0.017, *HLA-DRB6 p* = 0.003), tumor number (*HLA-DQA1 p* = 0.029, *HLA-DRA p* = 0.029), tumor size (*HLA-DQA2 p* = 0.047, *HLA-DQB2 p* = 0.047, *HLA-DRB6 p* = 0.011), Edmonson most grade (*HLA-DOB*, *p* = 0.003 *HLA-DQA2 p* = 0.007, *HLA-DQB2 p* = 0.014) and cirrhosis (*HLA-DOB p* = 0.034, *HLA-DQA1 p* = 0.008, *HLA-DRB1 p* = 0.034). According to survival analysis results *HLA-DMA* (*p* = 0.011), *HLA-DMB* (*p* = 0.027), *HLA-DOA* (*p =* 0.025), *HLA-DOB* (*p* = 0.002), *HLA-DPB1* (*p* = 0.047), *HLA-DQA2* (*p* = 0.004), *HLA-DQB2* (*p* = 0.021) and *HLA-DRB6* (*p* = 0.006) were significantly associated with better disease-free survival status (Table S3).

### 3.2. Clinical Analysis of HLA Genes (Validation Cohort)

Association between HLA genes and clinicopathological factors in the validation cohort is summarized in Table S4. According to the results, elevated levels of MHC class 1 genes including *HLA-C*, *HLA-H*, *HLA-E*, *HLA-F*, *HLA-G*, and *HLA-H* were associated with HCV (*HLA-C p* = 0.002, *HLA-E p* = 0.005, *HLA-F p* = 0.000, *HLA-G p* = 0.009, *HLA-H p* = 0.007). *HLA-C* showed an association with sex, alcohol consumption, cirrhosis with *p* values = 0.001, 0.045, 0.043 respectively. Of note, the *HLA-H* gene showed maximum association with clinical parameters including age (*p* = 0.048), sex (*p* = 0.023), tumor-stage (0.004) and T stage (*p* = 0.002). *HLA-G* (*p* = 0.007) and *HLA-E* (*p* = 0.003) also showed association with disease recurrence. Moreover, survival curves demonstrated that patients with high levels of *HLA-E* (*p* = 0.034) and *HLA-H* (0.045) were significantly associated with improved survival (Table S4). In addition, MHC class II genes including *HLA-DMA* (*p* = 0.003), *HLA-DMB* (*p* = 0.027), *HLA-DOB* (*p* = 0.005), *HLA-DRB1* (*p* = 0.027) and *HLA-DRB5* (*p* = 0.019) showed significant association with alcohol (Table S4). Elevated levels of *HLA-DOB* (*p* = 0.014) and *HLA-DQA2* (*p* = 0.049) were significantly associated with T-stage <2. *HLA-DOA* (*p* = 0.033), *HLA-DQA2* (*p* = 0.032), *HLA-DRA* (*p* = 0.038) and *HLA-DOB* (*p* = 0.005) showed association with initial tumor stage. Kaplan–Meier survival analysis revealed that high expression of *HLA-DOA* (*p* = 0.038), *HLA-DMA* (*p* = 0.022), *HLA-DMB* (*p* = 0.005), *HLA-DRA* (*p* = 0.006), *HLA-DRB1* (*p* = 0.018) and *HLA-DRB6* (*p* = 0.016) were associated with delayed recurrence (Table S4). Interestingly, four genes of MHC Class II *HLA-DOA*, *HLA-DMA*, *HLA-DMB* and *HLA-DRB6* showed significant association with disease free survival in both cohorts.

### 3.3. Clustering Analysis of HLA Genes with Adaptive Immune and HCC Related Genes

Out of a total 822 genes, expression levels of 505 genes were identified in both cohorts. Hierarchical clustering was performed on both cohorts using normalized expression of 505 genes by applying a heatmap function and ward d2 clustering method. Significant clusters of genes were selected which showed possible co-expression with *HLAs* (Figure S1). Selected clusters were further validated using the Spearman correlation method (Table S5).

Lastly, out of all these genes, a set of 40 unique genes showing positive correlation with HLAs were selected for further analysis (Table S6).

### 3.4. Network Analysis and Hub Gene Identification of 40 Genes Co-Expressed with HLAs 

Next, gene–gene interaction among 40 unique genes and HLAs were obtained from GeneMania. The gene–gene interaction network showed interactions based on various parameters where co-expression based interactions (in light purple color) were 72.63%, physical interactions among the genes scored 6.40% (marked in pink color) of all interactions, genetic interactions (in light green color) were 5.60% and the rest were the rare interactions (Figure S2).

Furthermore, degree centrality, betweenness, closeness, bottleneck and MCC calculation methods were chosen as the measure of hub node identification in CytoHubba. Network results showed the top 10 genes as potential hub nodes (Figure S3). Common genes present in all five networks including *NCF4*, *TYROBP*, *LCP2*, *ZAP70*, *PTPRC*, *FYN* and *WAS* were identified as key hub nodes.

### 3.5. Clinical Analysis of Seven Genes Signature

**Figure 2:**
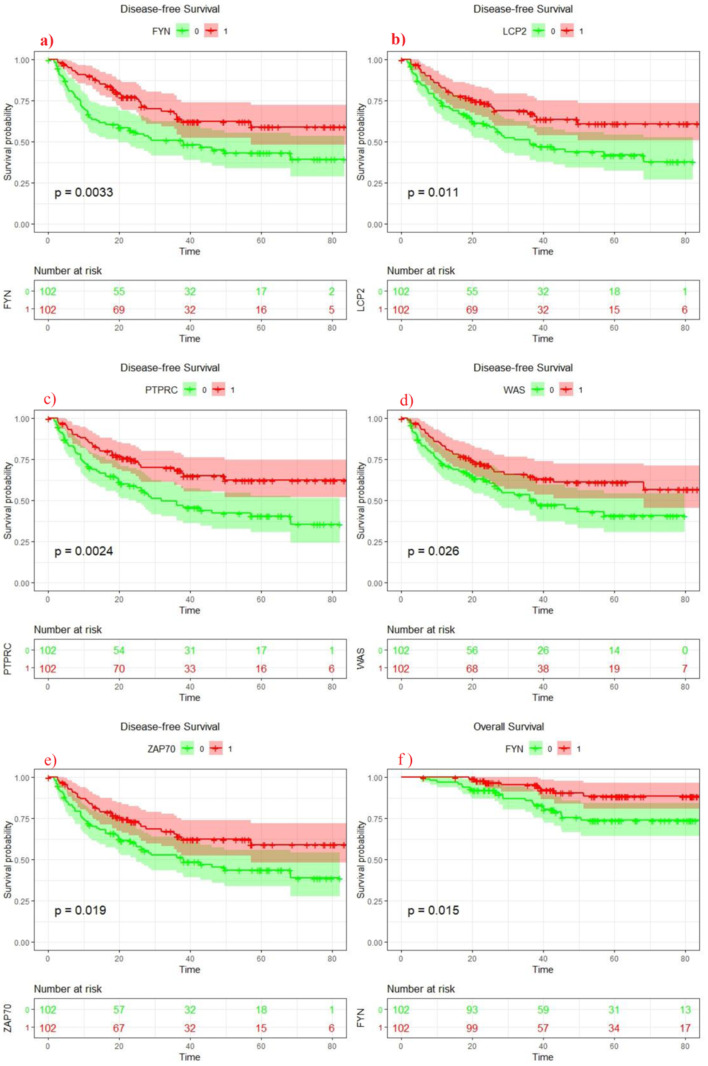
Kaplan–Meier estimates of disease-free survival of (**a**): FYN, (**b**): LCP2, (**c**): PTPRC, (**d**): WAS, (**e**): ZAP70 expression in HCC patients (Discovery cohort). (**f**): Kaplan–Meier estimates of overall survival of FYN gene (Discovery cohort).

Next, the clinical relevance of these seven HLA associated genes was evaluated. All these genes showed significant associations with different clinical features (Table 1). Upregulation of *TYROBP* and *WAS* showed significant association with age <60 (*p* value = 0.035). Elevated expression level of *FYN* (*p* value = 0.000), *LCP2* (*p* value = 0.002), *PTPRC* (*p* value = 0.011), *WAS* (*p* value = 0.005), *ZAP70* (*p* values = 0.000) and *NCF4* (*p* values = 0.047) were associated with tumor size. *TYROBP* (*p* values = 0.029) and *NCF4* (*p* values = 0.003) were also significantly associated with Edmonson most grade. Kaplan–Meier survival results showed that upregulation of *LCP2*, *PTPRC*, *ZAP70*, *FYN* and *WAS* was significantly associated with increased disease-free survival. In addition, elevated expression of only *FYN* was associated with better overall survival (Figure 2).

**Figure 3:**
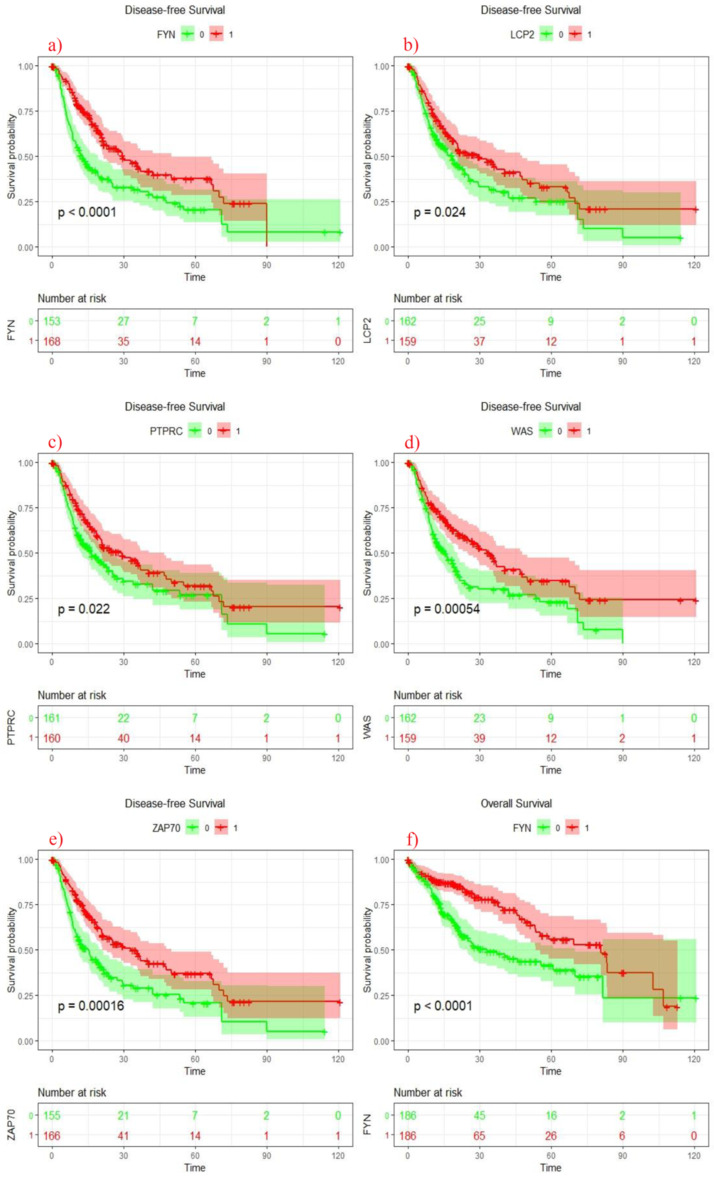
Kaplan–Meier estimates of disease-free survival of (**a**): FYN, (**b**): LCP2 (**c**): PTPRC, (**d**): WAS, (**e**): ZAP70 expression in HCC patients (Validation cohort) (**f**): Kaplan–Meier estimates of overall survival of FYN expression in HCC patients (Validation cohort).

Similarly, in validation cohort, high expression of these genes showed significant association with different clinical features including sex *(FYN p* = 0.038), grade (*FYN p* = 0.001), tumor stage *WAS p* = 0.037, *ZAP70 p* = 0.043) and T stage (*WAS p* = 0.049, *ZAP70 p* = 0.049) (Table 2). Kaplan–Meier survival curves results showed that elevated expression of *LCP2*, *PTPRC*, *WAS*, *ZAP70* and *FYN* were associated with delayed recurrence. Furthermore, upregulation of *FYN* and *ZAP70* showed significant association with better survival (Figure 3).

### 3.6. Survival Analysis of 7 Genes Signature Score

**Figure 4:**
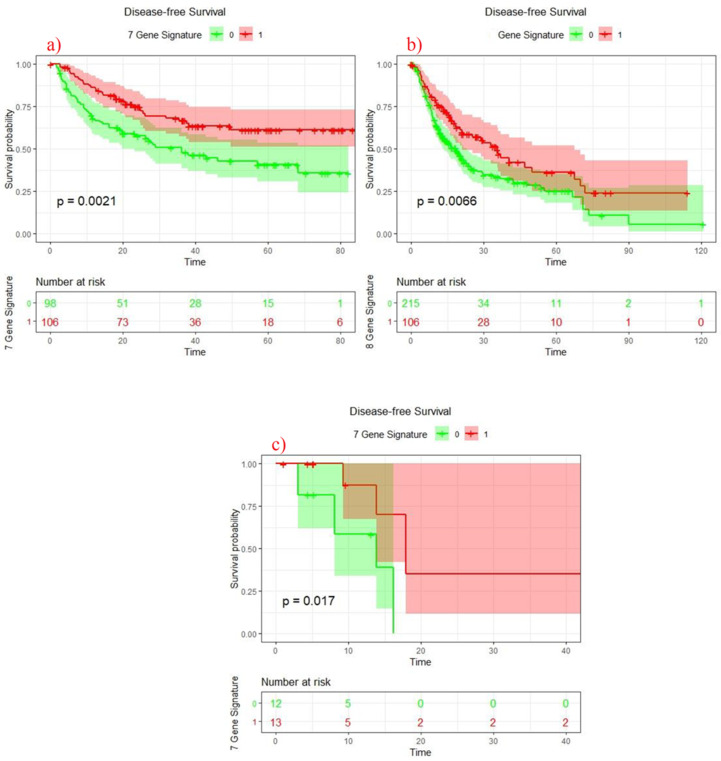
Survival analysis of seven genes signature ((**a**): Discovery cohort, (**b**): Validation cohort and (**c**): GSE45114 dataset).

Lastly, after identifying the significance of these seven MHC linked genes at multiple genetic and prognostic levels, we generated a score based on the mean expression of all significant genes. Interestingly, Kaplan–Meier survival analysis revealed that upregulation of seven gene signature in both cohorts (Discovery *p* = 0.0021) and (Validation *p* = 0.0066) showed improved disease-free survival in HCC patients (Figure 4a,b). Of note, we also downloaded another HCC expression cohort (ID: GSE45114) to further establish the association between seven gene signature and disease recurrence. Interestingly, after gene signature score estimation, we identified that upregulation of seven gene signature showed association with increased DFS of HCC patients (*p* = 0.017) (Figure 4c).

### 3.7. COX Regression Survival Analysis in Discovery and Validation Cohorts

Next, to further evaluate whether 7-gene signature may act as an independent biomarker for DFS, cox regression analysis was performed. According to multivariate analysis results of discovery cohort, disease free survival was positively associated (i.e., Hazard Ratio (HR) < 1) with 7-gene signature (HR 0.454, CI 0.291–0.710, *p* = 0.001), AFP (HR 0.573, CI 0.352–0.935, *p* = 0.026), while negatively associated (i.e., HR > 1) with tumor number (HR 5.895, CI 2.621–13.259, *p* = 0.000) (Table 3). Furthermore, according to multivariate analysis results validation cohort, 7-gene signature (HR 0.598, CI 0.390–0.917, *p* = 0.018) and HBV (HR 0.608, CI 0.403–0.920, *p* = 0.018) were positively associated with DFS, while tumor stage (HR 1.874, CI 1.248–2.814, *p* = 0.002) was negatively associated with DFS of HCC patients (Table 4).

Overall, the results depicted that HLA genes are significantly associated with delayed tumor recurrence. In addition, HLA linked gene signature was found to be an independent prognostic marker for delayed recurrence in three independent HCC datasets.

## 4. Discussion

Clinically, HCC is one of the most challenging cancers with an increasing mortality rate. In order to improve patient prognosis, the focus of HCC is gradually transforming from treatment to boosting host immune response [24,25]. Hence, identification of potential biomarkers for devising new immunotherapies and boosting patient prognosis are the prime focus of the study. In this study, using RNA-seq data of 576 HCC patients, a panel of seven genes (including LCP2, TYROBP, ZAP70, PTPRC, FYN, WAS and NCF4) has been identified which are independent predictors of delayed tumor recurrence and improved patient prognosis.

According to the statistical analysis results, several HLA genes (including HLA-A, HLA-C, HLA-E, HLA-F, HLA-G, HLA-L, HLA-DMB, HLA-DQA2 and HLA-DRA) showed association with different clinicopathological features in both HCC cohorts. Recent studies demonstrated the linkage of all MHC classes with HCC prognosis [26]. Interestingly, according to our results, HLA-E, HLA-DMA, HLA-DMB, HLA-DOA and HLA-DRB6 showed association with improved disease-free survival in both cohorts, emphasizing the need to investigate the functional implications of HLA expression in HCC using pre-clinical and clinical studies.

Interestingly, our study showed strong association of five HLA genes (including HLA-DMA, HLA-DMB, HLA-DOA, HLA-DRB6 and HLA-E) with delayed tumor recurrence in both cohorts. Previous studies have demonstrated aberrant expression of HLA class II components in epithelial ovarian cancer cells [27]. Consistent with our findings, HLA-DMB expression in ovarian cancer epithelial cells was associated with CD8 T cell infiltration and a marked improvement in disease survival [27]. Similarly, a recent study showed decreased expression of HLA-DMA, HLA-DMB and HLA-DOA in HBV associated HCC patients [26]. In a recent study, HLA-DRB6 was found to be upregulated among cervical cancer tissues and cell line data [28]. Additionally, a decreased expression of HLA-DRB6 was also reported in HCC patients compared to non-tumor liver tissues [26,29]. Furthermore, a cell line based study suggested that the upregulation of HLA-E represents a mechanism of tumor escape from NK cytotoxicity in ovarian cancer and melanoma [30]. Additionally, dysregulation of HLA-E expression and genetic mutations are also previously reported in HCC. However, the clear mechanism of how HLA-E affects tumor environment is largely unknown.

Recently, reactivation of the T cell receptor (TCR) signaling pathway gained focus in HCC [31,32]. Interestingly, most of the HLA linked genes identified in our study work in concordance to modulate TCR signaling, except *NCF4*. For instance, activation of CD3 heterodimer complex (TCR-CD3) on T cell membrane stimulates *ZAP70* responsible for both recruitment and phosphorylation of numerous downstream adaptors proteins. Two main substrates for *ZAP70* are linked for the activation of T cells (LAT) and LCP-2/SLP-76 [33]. Interestingly, *ZAP70* influences calcium mobilization (via PL-Cγ1) and ras activation (via GRB2) by interacting with LAT, while for cytoskeleton rearrangement (via VAV1), LCP-2 complex plays a pivotal role. Regulation of these signaling cascades sets the threshold of activation of naive T cells so that they are not activated by self-peptide-MHC complexes but respond only to foreign peptide–MHC complex [34]. This signaling triggers Rho family GTPases (Rac1 and Rac2), leading to actin polymerization via Wiskott–Aldrich syndrome protein (WASP) and ARP2-ARP3 complex. This structural rearrangement is desired for both recognition and induction of cytotoxic effect on APC cells. Hence, enhanced *LCP-2* significantly boosts cell mediated immune response. Involvement of *LCP-2* as the key mediator responsible for enhanced T cell activation and immune function is already known [35].

Additionally, several co-stimulatory factors also regulate maturation of naive T-cells. TCR-CD3 complex also activates Src family kinase members (Lck and Fyn), leading to activation of several signaling pathways, such as the ERK pathway [36]. Interestingly, *FYN* irrespective of Lck can also stimulate the ERK pathway while PTPRC (CD45) maintains dephosphorylated form at Tyr^505^ of C-terminal residues of Lck and Fyn retaining their active conformation inside cell [37]. Of note, *TYROBP* is also previously identified as a substrate of *PTPRC* gene in TCR signaling [38].

Of note, these seven genes were linked with different cancers in previous studies. For instance, *LCP2* expression was linked with improved survival in breast and ovarian cancer [39]. Similarly, *ZAP70*, *PTPRC*, *FYN* and *WASP2* were found to be dysregulated in different cancers including breast, leukemia, colorectal cancer, etc. [40,41,42,43]. However, none of the studies showed any linkage of these seven genes with delayed tumor recurrence in HCC. Our integrated approach provided a novel insight in predicting new prognostic subtypes of HCC. However, due to the sparsity of the high throughput sequencing data and mixture of HBV and HCV infected patients, further in-vitro and in-vivo studies are warranted to evaluate the effect of HBV/HCV infection before consideration of gene signature in clinical practice.

In conclusion, our data suggests that the seven gene signature can boost immune response in HCC patients by mainly regulating TCR signaling. Earlier studies showed that TCR associated immunotherapies are also emerging as new treatment options against HCC. Interestingly, we demonstrated that despite the substantial heterogeneity of HCC at the genomic level, whole transcriptomic data analysis is an excellent strategy to identify new biomarkers for improving HCC prognosis. Of note, this immune related signature is also validated in three independent cohorts, suggesting it as a potential biomarker for HCC patients worldwide.

## Figures and Tables

**Table 1:** Association of expression of 7 genes signature with clinical features in Discovery cohort.

Gene ID	Age (Age < 60)	Tumor Size (Size < 5)	Edmonson Grade (Advance)	Microvascular Invasion (Negative)	AFP Level	Overall Survival (Better)	Disease Free Survival (Delayed)
*FYN*	-	Upregulation	-	Upregulation	Upregulation(High)	Upregulation -	Upregulation
*LCP2*	-	Upregulation	-	Upregulation	-	-	Upregulation
*NCF4*	-	Upregulation	Upregulation	-	-	-	-
*PTPRC*	-	Upregulation	-	Upregulation	Upregulation(Low)	-	Upregulation
*TYROBP*	Upregulation	-	Upregulation	-	-	-	-
*WAS*	Upregulation	Upregulation	-	-	-	-	Upregulation
*ZAP70*	-	Upregulation	-	Upregulation	Upregulation(Low)	-	Upregulation

**Table 2:** Association of expression of seven genes signature with clinical features (Validation cohort).

Gene ID	Sex (Male)	Grade (Early)	HCV (Negative)	AFP Level (Low)	Alcohol Consumption (High)	Tumor Stages (Early)	Overall Survival (Better)	Disease Free Survival (Delayed)
*FYN*	Upregulation	Upregulation	-	Upregulation	-	-	Upregulation-	Upregulation
*LCP2*	-	-	-	-	-	-	-	Upregulation
*NCF4*	-	-	Upregulation	-	Upregulation	-	-	-
*PTPRC*	-	-	-	-	-	-	-	Upregulation
*TYROBP*	-	-	-	-	Upregulation	-	-	-
*WAS*	-	-	Upregulation	-	Upregulation	Upregulation	-	Upregulation
*ZAP70*	-	-	Upregulation	-	Upregulation	Upregulation	Upregulation	Upregulation

**Table 3:** Cox Regression Analysis for disease free survival (Discovery cohort).

Univariate Analysis								Multivariate Analysis						
	B	SE	Wald	Sig.	Exp(B)	95.0% CI for Exp(B)		B	SE	Wald	Sig.	HR	95.0% CI for Exp(B)	
						Lower	Upper						Lower	Upper
7GeneSignature	−0.665	0.221	9.075	0.003	0.514	0.334	0.793	−0.789	0.228	11.983	0.001	0.454	0.291	0.710
AFP high/low	−0.509	0.238	4.584	0.032	0.601	0.377	0.958	−0.556	0.249	4.980	0.026	0.573	0.352	0.935
Size	0.461	0.222	4.298	0.038	1.586	1.026	2.453	-	-	-	-	-	-	-
Tumor Number	1.440	0.375	14.710	0.000	4.221	2.022	8.810	1.774	0.414	18.403	0.000	5.895	2.621	13.259

**Table 4:** Cox Regression Analysis for disease free survival (Validation cohort).

Univariate Analysis								Multivariate Analysis						
	B	SE	Wald	Sig.	Exp(B)	95.0% CI for Exp(B)		B	SE	Wald	Sig.	HR	95.0% CI for Exp(B)	
						Lower	Upper						Lower	Upper
7GeneSignature	−0.450	0.167	7.247	0.007	0.638	0.460	0.885	−0.514	0.218	5.563	0.018	0.598	0.390	0.917
HBV	−0.664	0.180	13.592	0.000	0.515	0.361	0.733	−0.497	0.211	5.561	0.018	0.608	0.403	0.920
Metastasis	1.581	0.592	7.134	0.008	4.862	1.523	15.516	-	-	-	-	-	-	-
T-stage	0.834	0.163	26.038	0.000	2.302	1.671	3.170	0.628	0.207	9.181	0.002	1.874	1.248	2.814

## Data Availability

Multiple publicly available datasets are used in the study. The details are provided in the methodology section where necessary.

## Supplementary Materials

The following are available online at https://www.mdpi.com/article/10.3390/jpm11111129/s1, Figure S1: Hierarchical clustering of 505 gene signature in discovery and validation cohorts of HCC patients based on upregulation and downregulation of HLA-DMA, HLA-DMB, HLA-DRB6, HLA-DOA, HLA-E (colored bar red for upregulation and yellow for downregulation), Figure S2: GeneMania interaction network of 40 genes signature and HLA-DMA, HLA-DMB, HLA-DRB6, HLA-DOA, HLA-E with co-expression, colocalization and pathways, Figure S3: Network of 40 genes signature and HLA-DMA, HLA-DMB, HLA-DRB6, HLA-DOA, HLA-E. Network shows top 10 hub genes based on (A): Degree measure, (B): MCC, (C): Betweenness, (D): Closeness, (E): Bottleneck, Table S1: Clinicopathological characteristics of Discovery cohort (204 HCC patients), Table S2: Clinicopathological characteristics of Validation cohort (372 HCC patients), Table S3: Association of expression of HLA genes with clinical features in Discovery cohort, Table S4: Association of expression of HLA genes with clinical features in Validation cohort, Table S5: MHC gene and their correlated gene list, Table S6: Gene name and chromosomal location of 40 unique genes.
