# Supplementary material for: Effect of MHC Linked 7-Gene Signature on Delayed Hepatocellular Carcinoma Recurrence
Source: J Pers Med. 2021 Nov 2;11(11):1129. doi: 10.3390/jpm11111129 (PMC8625636; doi:10.3390/jpm11111129)

1A. Discovery and Validation datasets of HLA-DMA

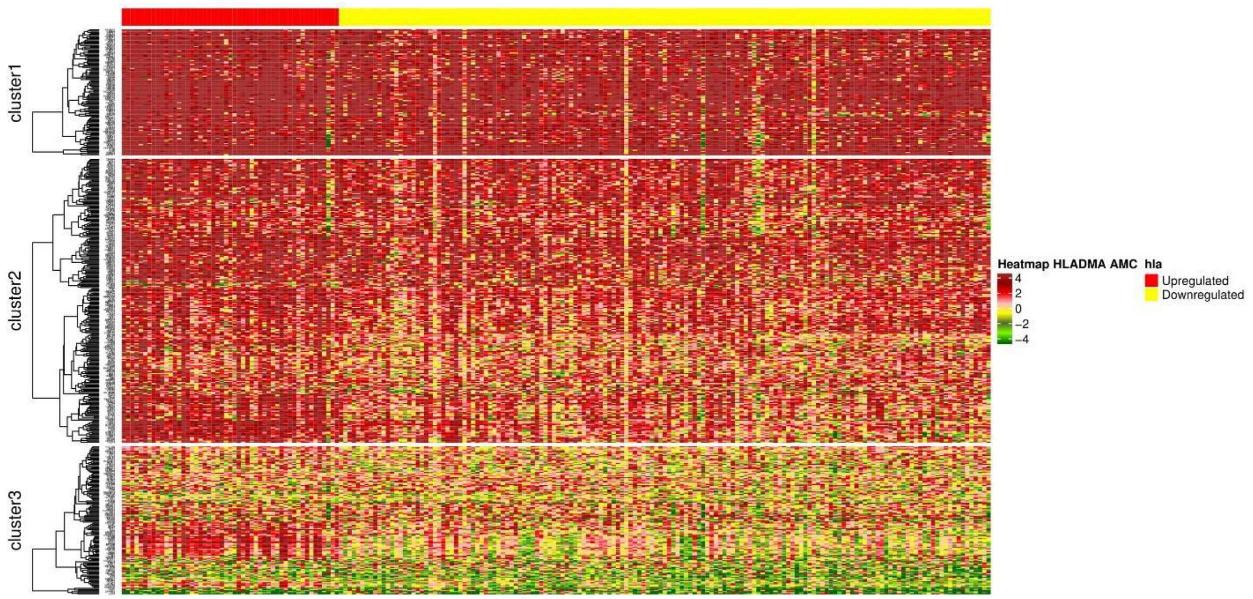

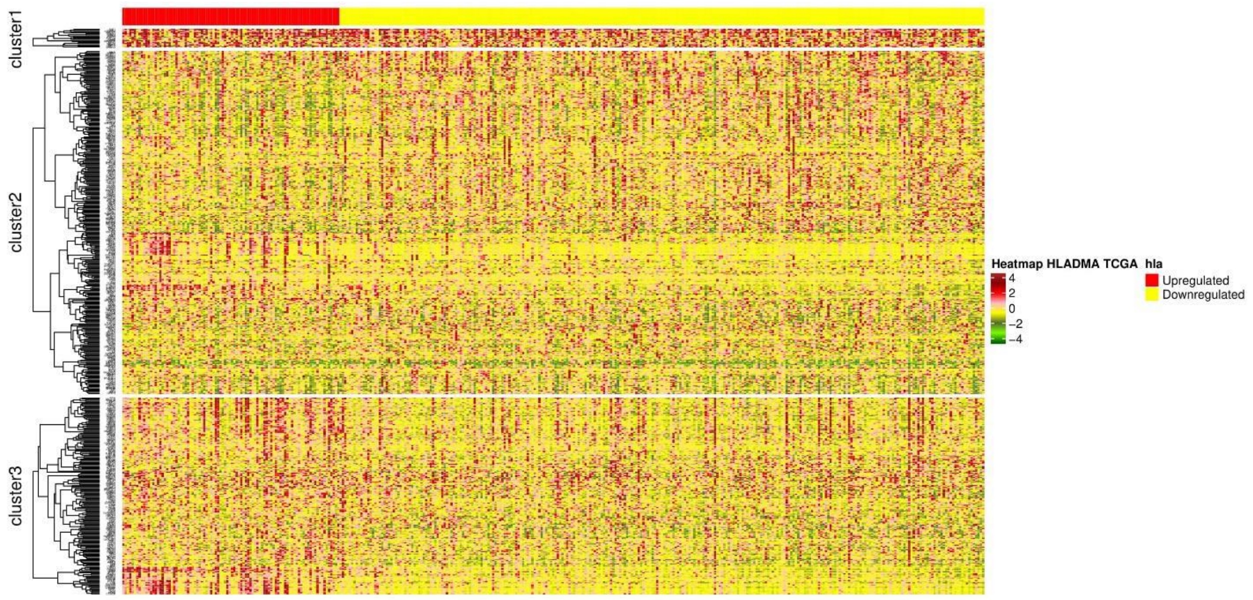

1B. Discovery and Validation datasets of HLA-DMB

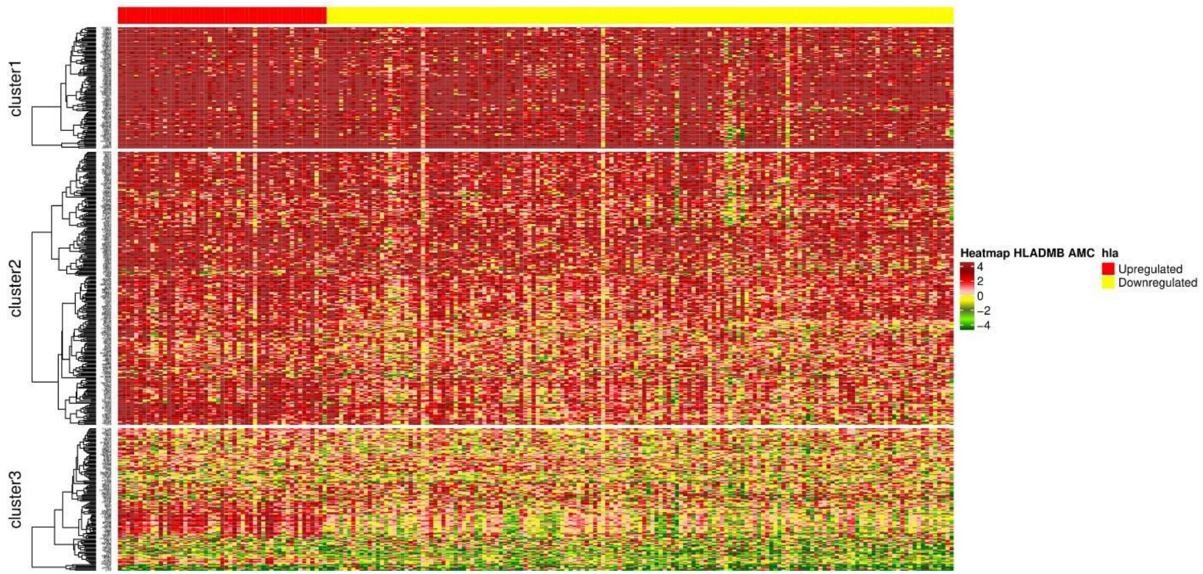

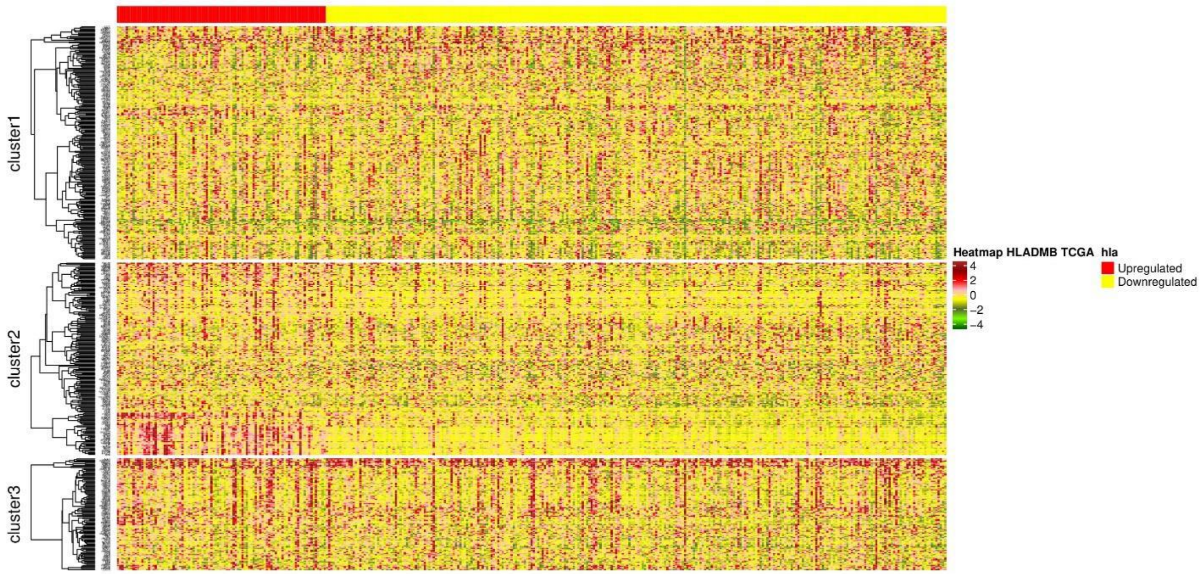

1C. Discovery and Validation datasets of HLA-DOA

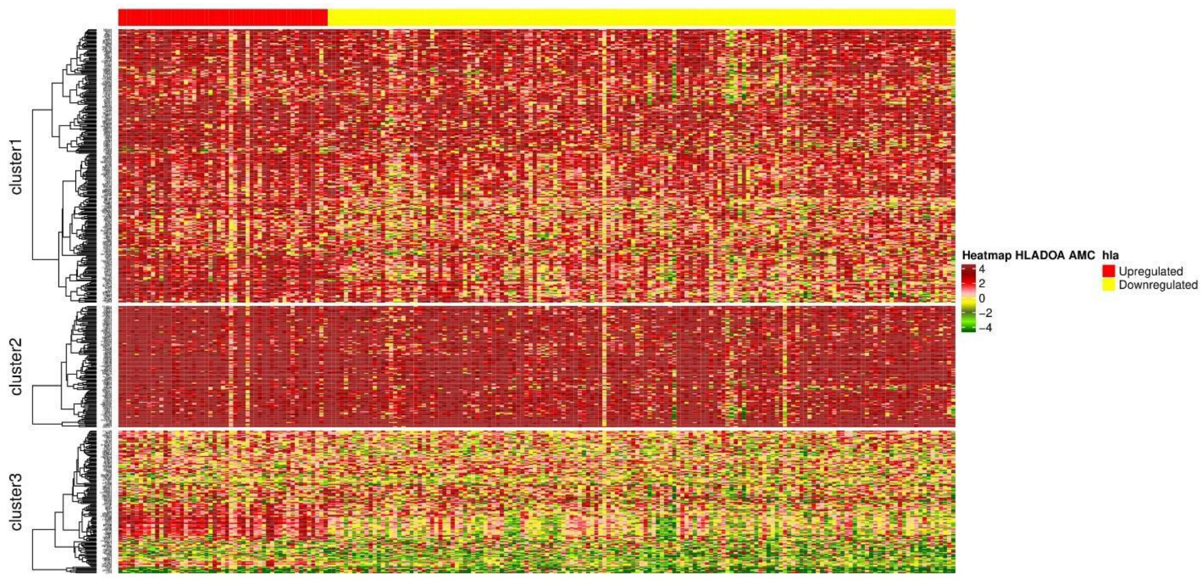

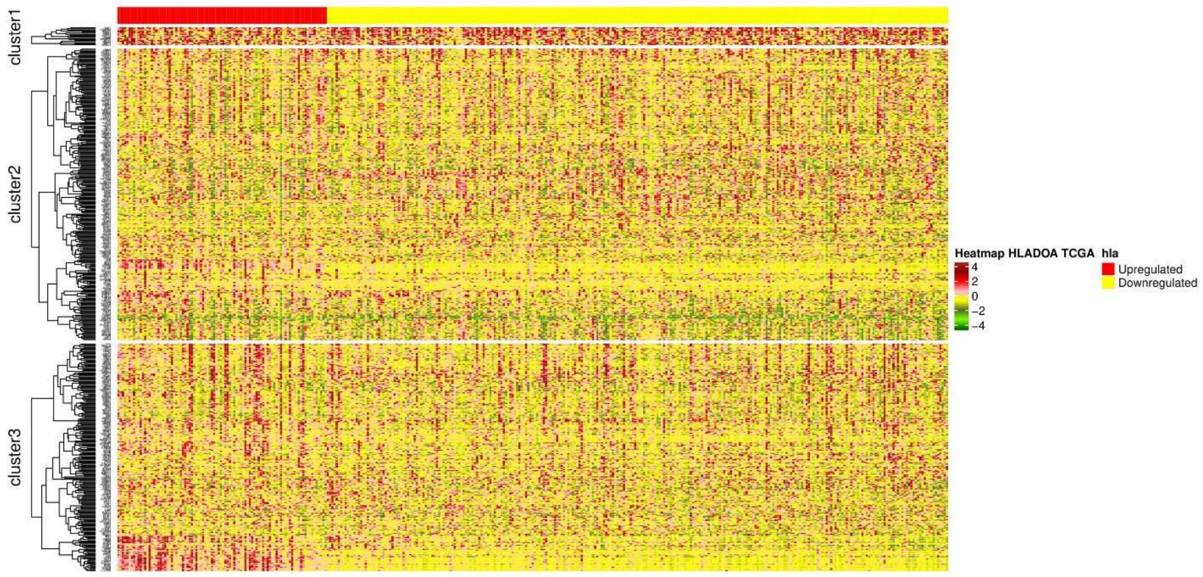

1C. Discovery and Validation datasets of HLA-DRB6

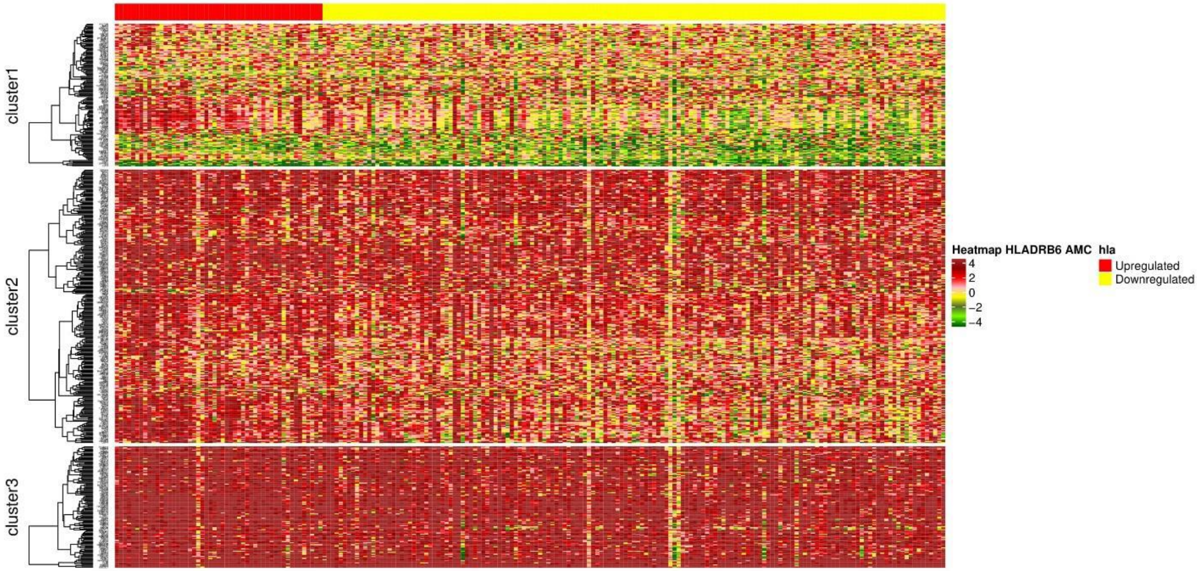

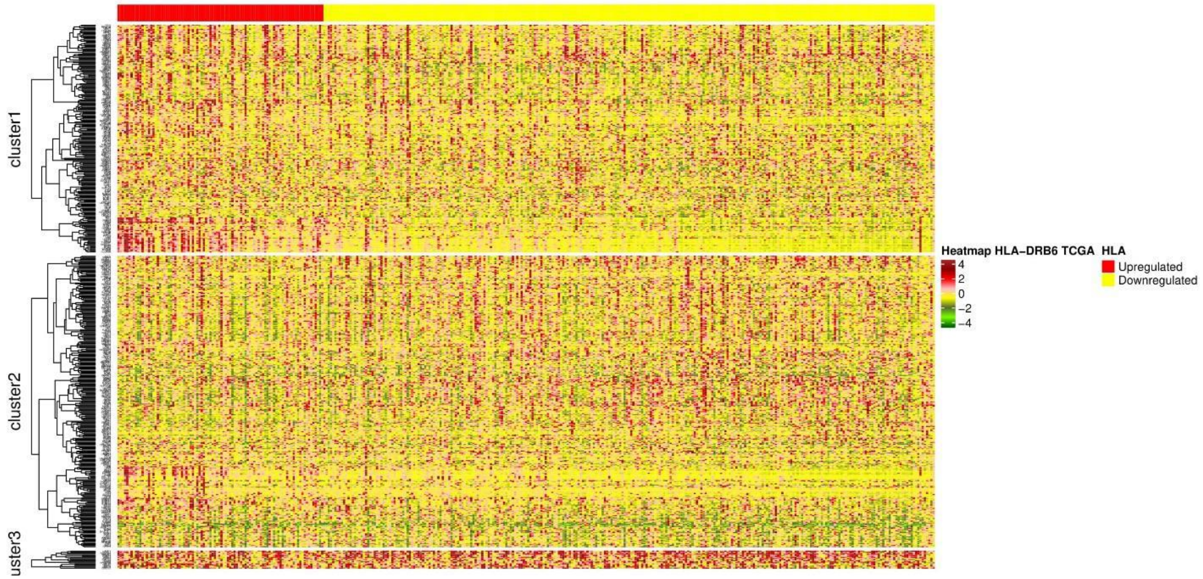

1C. Discovery and Validation datasets of HLA-E

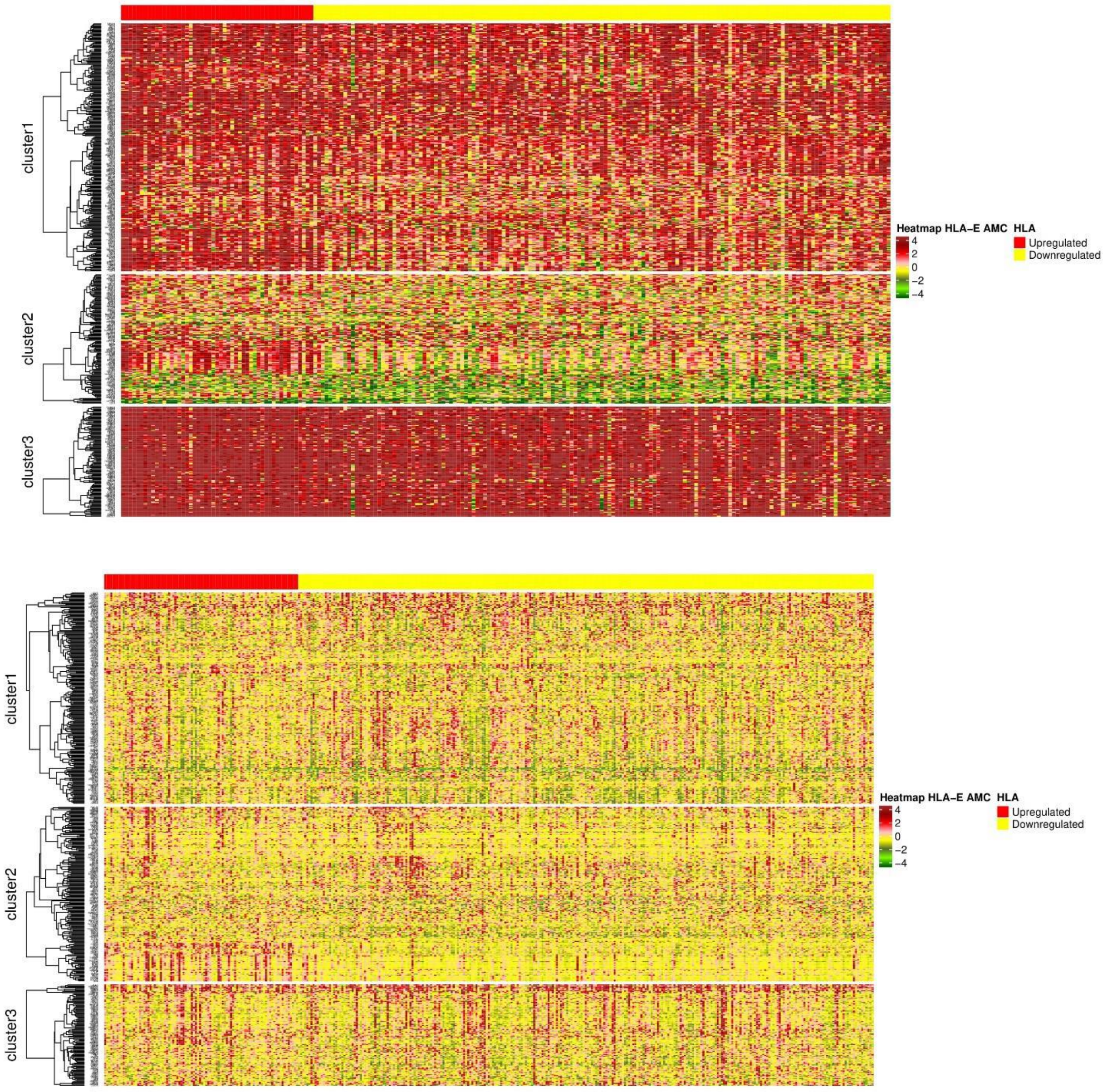

Supplement: Supplementary file 1 [file jpm-11-01129-s001.zip › Figure S1.pdf]
