# Supplementary material for: Effect of MHC Linked 7-Gene Signature on Delayed Hepatocellular Carcinoma Recurrence
Source: J Pers Med. 2021 Nov 2;11(11):1129. doi: 10.3390/jpm11111129 (PMC8625636; doi:10.3390/jpm11111129)
Supplement: Supplementary file 1 [file jpm-11-01129-s001.zip › Figure S2.pdf]

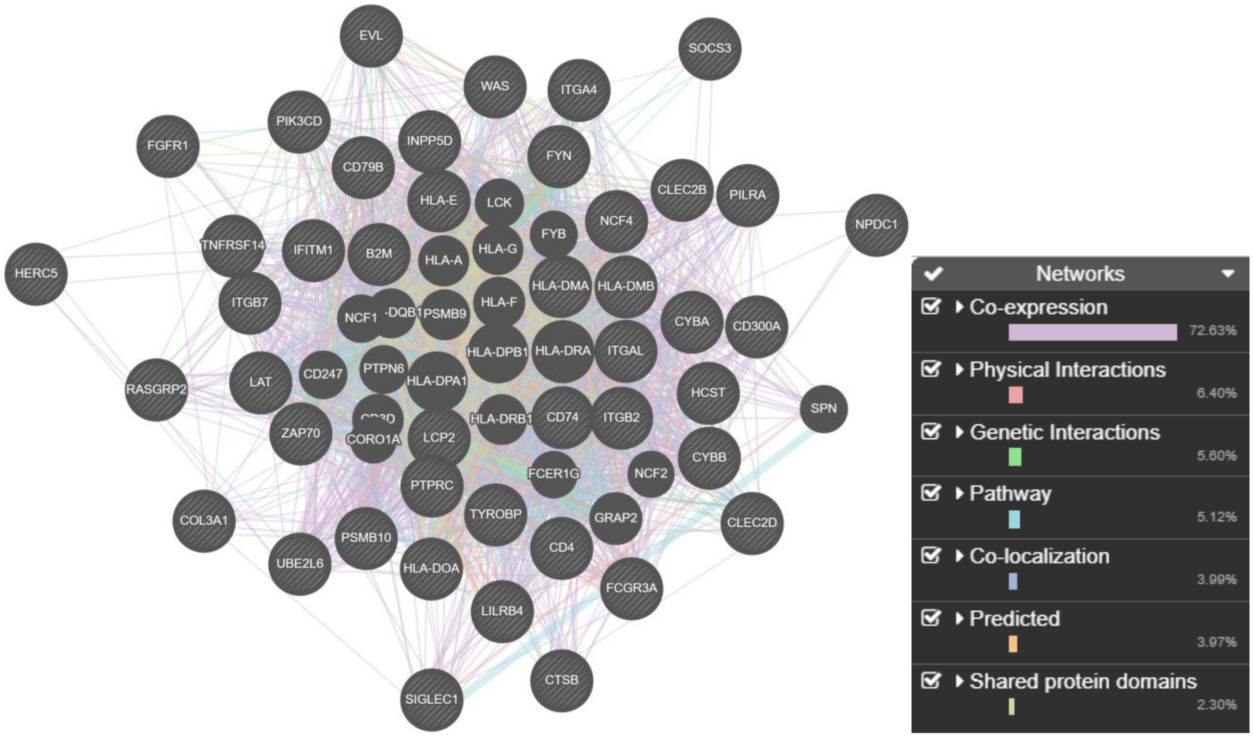
EVL
SOCS3
WAS
ITGA4
PIK3CD
FGFR1
INPP5D
CD79B
FYN
CLEC2B
PILRA
NPDC1
HLA-E
LCK
NCF4
TNFRSF14
IFITM1
B2M
HLA-A
HLA-G
FYB
HERC5
ITGB7
NCF1
-DQB1
PSMB9
HLA-F
HLA-DMA
HLA-DMB
CYBA
CD300A
HLA-DPB1
HLA-DRA
ITGAL
RASGRP2
LAT
CD247
PTPN6
HLA-DPA1
HCST
SPN
ZAP70
CORO1A
LCP2
HLA-DRB1
CD74
ITGB2
CYBB
FCER1G
NCF2
PTPRC
TYROBP
COL3A1
GRAP2
CLEC2D
PSMB10
CD4
UBE2L6
HLA-DOA
FCGR3A
LILRB4
CTSB
SIGLEC1
Networks
Co-expression
72.63%
Physical Interactions
6.40%
Genetic Interactions
5.60%
Pathway
5.12%
Co-localization
3.99%
Predicted
3.97%
Shared protein domains
2.30%
