# Supplementary material for: Effect of MHC Linked 7-Gene Signature on Delayed Hepatocellular Carcinoma Recurrence
Source: J Pers Med. 2021 Nov 2;11(11):1129. doi: 10.3390/jpm11111129 (PMC8625636; doi:10.3390/jpm11111129)
Supplement: Supplementary file 1 [file jpm-11-01129-s001.zip › jpm-1407072-supplementary.pdf]

Supplementary Material

Supplementary Tables:

Table S1. Clinicopathological characteristics of Discovery cohort (204 HCC patients)

Characteristics	Total Patients=N=204
**Diagnosis Age**	
**<60years**	139(68.14%)
**>=60years**	65(31.86%)
**Sex**	
**Female**	152(74.51%)
**Male**	52(25.49%)
**HBV vs non-HBV**	
**HBV**	148(72.55%)
**non-HBV**	56(27.45%)
**Tumor size**	
**<=5**	142(69.61%)
**>5**	62(30.39%)
**Edmonson most Grade**	
**<=2**	130(63.73%)
**>2**	74(36.27%)
**Alpha-fetoprotein level**	
**<10**	127(62.25%)
**>=10**	77(37.75%)
**Microvascular invasion**	
**No**	147(72.06%)
**Yes**	57(27.94%)
**Cirrhosis**	
**No**	106(51.96%)
**Yes**	98(48.04%)
**Overall survival**	
**Alive**	175(85.78%)
**Death**	29(14.22%)
**Disease Free survival**	
**Disease Free**	118(57.84%)
**Recurrence**	86(42.16%)

Table S2. Clinicopathological characteristics of Validation cohort (372 HCC patients)

Characteristics	Total patients=N=372
**Diagnosis Age**	
**<60years**	169(45.43%)
**>=60years**	203(54.57%)
**Sex**	
**Female**	121(32.53%)
**Male**	251(67.47%)
**HBV**	
**No**	248(66.67%)
**Yes**	105(28.23%)
**NA**	19
**HCV**	
**No**	297(79.84%)
**Yes**	56(15.05%)
**NA**	19
**HBV or HCV**	
**No**	199(53.49%)
**Yes**	154(41.40%)
**NA**	19
**HBV and HCV**	
**No**	346(93.01%)
**Yes**	7(1.88%)
**NA**	19
**Alcohol consumption**	
**No**	236(63.44%)
**Yes**	117(31.45%)
**NA**	19
**Grade**	
**G1-G2**	233(62.63%)
**G3-G4**	134(36.02%)
**NA**	5
**T stage**	
**T1-T2**	276(74.19%)
**T3-T4**	93(25%)
**NA**	3
**Residual tumor**	
**R0**	325(87.37%)
**R1-R2**	18(4.84%)
**NA**	29
**Alpha-fetoprotein level**	
**<200**	202(54.30%)
**>=200**	76(20.43%)
**NA**	94
**Node stage**	

**N0**	253(68.01%)
**N1**	4(1.07%)
**NA**	115
**Metastasis stage**	
**M0**	267(71.77%)
**M1**	4(1.08%)
**NA**	101
**Tumor stage**	
**Stage I-II**	258(69.35%)
**Stage III-IV**	90(24.19%)
**NA**	24
**Microvascular invasion**	
**No**	16(4.30%)
**Yes**	93(25%)
**NA**	263
**Macrovascular invasion**	
**No**	93(25%)
**Yes**	16(4.30%)
**NA**	263
**Cirrhosis**	
**Fibrosis**	68(18.28%)
**Cirrhosis**	70(18.81%)
**NA**	234
**Overall survival**	
**Alive**	242(65.05%)
**Death**	130(34.95%)
**Disease free survival**	
**Disease free**	145(38.98%)
**Recurrence**	176(47.31%)
**NA**	51

*NA = Not Available

Table S3. Association of expression of HLA genes with clinical features in Discovery cohort

Gene ID	Sex	Age	Tumor size	HBV vs non-HBV	Edmonson most grade	Cirrhosis	Alpha-fetoprotein level	Microvascular	Kaplan-Meier overall . ,	Kaplan-Meier disease
HLA-A	NA	NA	NA	NA	NA	NA	NA	NA	NA	NA
HLA-B	NA	NA	NA	NA	NA	NA	NA	NA	NA	NA
HLA-C		NA	NA	NA		NA		NA	NA	NA
HLA-DMA	NA	NA	NA	NA	NA	NA	NA	NA	NA	
HLA-DMB	NA		NA	NA	NA	NA	NA	NA	NA	
HLA-DOA		NA	NA	NA	NA	NA	NA	NA	NA	
HLA-DOB	NA	NA	NA	NA			NA	NA	NA	
HLA-DPA1	NA		NA	NA	NA	NA	NA	NA	NA	NA
HLA-DPB1	NA		NA	NA	NA	NA	NA	NA	NA	
HLA-DPB2	NA	NA	NA	NA	NA	NA	NA	NA	NA	NA
HLA-DQA1	NA	NA	NA	NA	NA		NA	NA	NA	NA
HLA-DQA2	NA			NA		NA	NA	NA		
HLA-DQB1	NA	NA	NA	NA	NA	NA	NA	NA	NA	NA
HLA-DQB2	NA			NA		NA	NA	NA	NA	
HLA-DRA	NA		NA	NA	NA	NA	NA	NA	NA	NA
HLA-DRB1	NA	NA	NA	NA	NA		NA	NA	NA	NA
HLA-DRB6	NA			NA	NA	NA	NA		NA	
HLA-E	NA	NA	NA			NA	NA	NA	NA	
HLA-F	NA	NA	NA	NA	NA		NA	NA	NA	NA
HLA-G		NA	NA	NA	NA	NA		NA		
HLA-H	NA	NA	NA	NA	NA	NA	NA	NA	NA	NA
HLA-J	NA	NA	NA	NA	NA	NA	NA	NA	NA	NA
HLA-L		NA		NA	NA	NA	NA	NA	NA	NA

Upregulation in Female, age<60, non-HBV, no Cirrhosis

Upregulation in Male, Advance grade stage, Tumor size>=5, Hight Alpha-fetoprotein level

Upregulation in no Microvascular invasion, Tumor size<5, Good survival,

Table S4. Association of expression of HLA genes with clinical features in Validation cohort

Gene ID	Age	Sex	Grade	HBV	HCV	HBV or HCV	HBV and HCV	Alpha-fetoprotein	Alcohol consumption	Cirrhosis	Tumor stage	T stage	Kaplan-Meier overall	Kaplan-Meier disease free
HLA-C	NA		NA	NA		NA	NA				NA	NA	NA	NA
HLA-DMA	NA	NA	NA	NA	NA	NA	NA	NA		NA	NA	NA	NA	
HLA-DMB	NA	NA	NA	NA	NA	NA	NA	NA		NA	NA	NA	NA	
HLA-DOA	NA	NA	NA	NA	NA	NA	NA	NA	NA	NA		NA	NA	
HLA-DOB	NA	NA	NA	NA			NA	NA		NA			NA	NA
HLA-DPB1	NA	NA	NA	NA	NA	NA	NA	NA	NA	NA	NA	NA	NA	NA
HLA-DPB2	NA		NA	NA	NA	NA	NA		NA	NA	NA	NA	NA	NA
HLA-DQA1	NA	NA	NA	NA	NA	NA	NA	NA	NA	NA	NA	NA	NA	NA
HLA-DQA2	NA	NA		NA		NA	NA	NA	NA	NA			NA	NA
HLA-DQB1	NA	NA	NA	NA	NA	NA	NA	NA	NA	NA	NA	NA	NA	NA
HLA-DQB2	NA	NA	NA	NA	NA	NA	NA	NA	NA	NA	NA	NA	NA	NA
HLA-DRA	NA	NA	NA	NA	NA	NA	NA	NA	NA	NA		NA	NA	
HLA-DRB1	NA	NA	NA	NA		NA	NA	NA		NA	NA	NA	NA	
HLA-DRB6	NA	NA	NA	NA	NA	NA	NA	NA	NA	NA	NA	NA	NA	
HLA-DRB5	NA	NA	NA	NA		NA	NA	NA		NA	NA	NA	NA	NA
HLA-E	NA	NA				NA	NA			NA	NA			
HLA-F		NA	NA	NA		NA			NA	NA			NA	NA
HLA-G	NA	NA	NA	NA			NA	NA	NA	NA	NA		NA	NA
HLA-H			NA	NA			NA	NA	NA	NA				NA

Upregulation in Female Initial grade stage,, non-HBV, non-HBV, no Cirrhosis, Initial T stage, , Low Alpha-fetoprotein level, non-HCV

Upregulation in Male, age>=60, Advance grade stage, HCV, Alcohol consumption, HBV or HCV, High Alpha-fetoprotein level, HBV and HCV

Upregulation in Initial tumor stage, Good survival, Delayed recurrence

NA: No Association

Table S5. MHC gene and their correlated gene list

MHC Gene name	Correlated Genes
**HLA-DMA**	CD300A, CD74, CD79B, CLEC2B, CYBA, HCST, IFITM1, LAT, LILRB4, NCF4, NPDC1, PILRA, PSMB10, RASGRP2, SIGLEC1, TYROBP, UBE2L6, WAS and TNFRSF14
**HLA-DMB**	B2M, CD300A, CD4, CD74, CD79B, CLEC2B, CLEC2D, COL3A1, CYBA, CYBB, EVL, FCGR3A, FYN, HCST, IFITM1, INPP5D, ITGA4, ITGAL, ITGB2, ITGB7, LAT, LCP2, LILRB4, NCF4, NPDC1, PIK3CD, PILRA, PTPRC, RASGRP2, SIGLEC1, SOCS3, TYROBP, UBE2L6, WAS, ZAP70
**HLA-DOA**	B2M, CD300A, CD74, CD79B, CLEC2B, CYBA, EVL, FCGR3A, HCST, IFITM1, ITGAL, ITGB2, LAT, LILRB4, NCF4, NPDC1, PIK3CD, PILRA, PSMB10, RASGRP2, SIGLEC1, TYROBP, UBE2L6, WAS, ZAP70
**HLA-DRB6**	B2M, CD300A, CD74, CD79B, CLEC2B, CYBA, FCGR3A, HCST, IFITM1, ITGAL, ITGB2, LILRB4, NCF4, PIK3CD, PILRA, PSMB10, RASGRP2, SIGLEC1, TYROBP, UBE2L6, WAS, ZAP70
**HLA-E**	B2M, CD74, UBE2L6, WAS, ITGB2, LCP2, PTPRC, PILRA, SIGLEC1, CYBB, FCGR3A, LILRB4, HERC5, IFITM1, NCF4, HCST, TYROBP, PIK3CD, CLEC2B, ZAP70, ITGA4, FYN, INPP5D, ITGAL, CD300A, CTSB, CD4, ITGB7, EVL, CD79B, SOCS3

Table S6. Gene name and chromosomal location of 40 unique genes

HGNC symbol	Chromosome location	Gene description
B2M	15q21.1	beta-2-microglobulin
CD300A	17q25.1	CD300a molecule
CD4	12p13.31	CD4 molecule
CD74	5q33.1	CD74 molecule
CD79B	17q23.3	CD79b molecule
CLEC2B	12p13.31	C-type lectin domain family 2 member B
CLEC2D	12p13.31	C-type lectin domain family 2 member D
COL3A1	2q32.2	collagen type III alpha 1 chain
CTSB	21q22.3	cathepsin B
CYBA	16q24.2	cytochrome b-245 alpha chain
CYBB	Xp21.1-p11.4	cytochrome b-245 beta chain
EVL	14q32.2	Enah/Vasp-like
FCGR3A	1q23.3	Fc fragment of IgG receptor IIIa
FGFR1	8p11.23	fibroblast growth factor receptor 1
FYN	6q21	FYN proto-oncogene, Src family tyrosine kinase
HCST	19q13.12	hematopoietic cell signal transducer
HERC5	4q22.1	HECT and RLD domain containing E3 ubiquitin protein ligase 5
IFITM1	11p15.5	interferon induced transmembrane protein 1
INPP5D	2q37.1	inositol polyphosphate-5-phosphatase D
ITGA4	2q31.3	integrin subunit alpha 4
ITGAL	16p11.2	integrin subunit alpha L
ITGB2	21q22.3	integrin subunit beta 2
ITGB7	12q13.13	integrin subunit beta 7
LAT	16p11.2	linker for activation of T cells
LCP2	5q35.1	lymphocyte cytosolic protein 2
LILRB4	19q13.4	leukocyte immunoglobulin like receptor B4
NCF4	22q12.3	neutrophil cytosolic factor 4
NPDC1	9q34.3	neural proliferation, differentiation and control 1
PIK3CD	1p36.22	phosphatidylinositol-4,5-bisphosphate 3-kinase catalytic subunit delta
PILRA	7q22.1	paired immunoglobin like type 2 receptor alpha
PSMB10	16q22.1	proteasome subunit beta 10
PTPRC	1q31.3	protein tyrosine phosphatase receptor type C
RASGRP2	11q13.1	RAS guanyl releasing protein 2
SIGLEC1	20p13	sialic acid binding Ig like lectin 1
SOCS3	17q25.3	suppressor of cytokine signaling 3
TNFRSF14	1p36.32	TNF receptor superfamily member 14
TYROBP	19q13.12	TYRO protein tyrosine kinase binding protein
UBE2L6	11q12.1	ubiquitin conjugating enzyme E2 L6

WAS	Xp11.23	WASP actin nucleation promoting factor
ZAP70	2q11.2	zeta chain of T cell receptor associated protein kinase 70
