# Supplementary figures and images for: Effect of MHC Linked 7-Gene Signature on Delayed Hepatocellular Carcinoma Recurrence
Source: J Pers Med. 2021 Nov 2;11(11):1129. doi: 10.3390/jpm11111129 (PMC8625636; doi:10.3390/jpm11111129)

Degree Measure

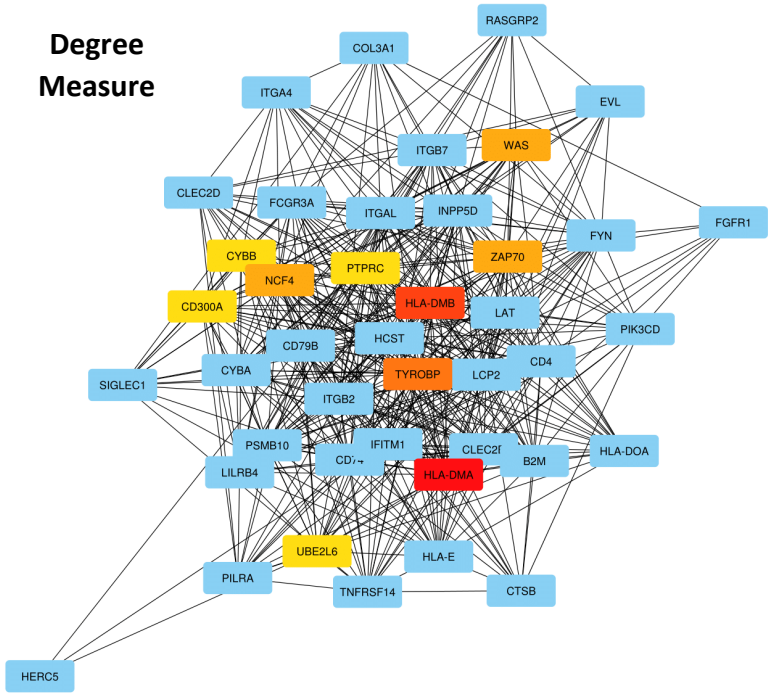

MCC Measure

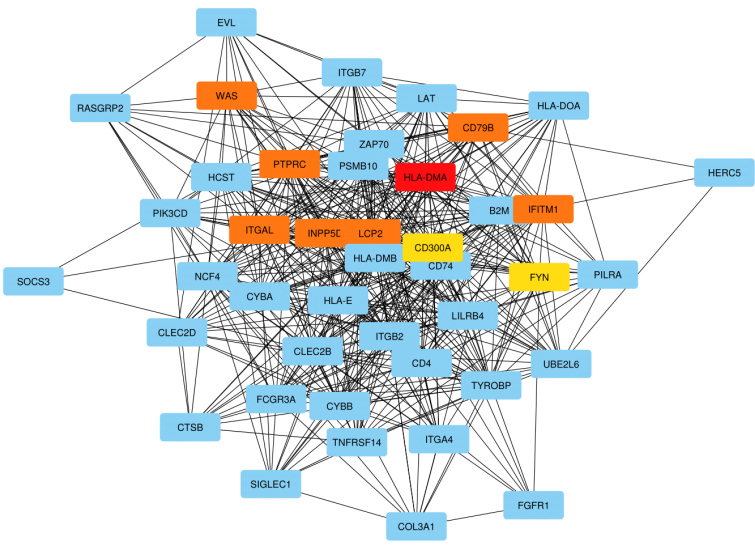

Betweenness Measure

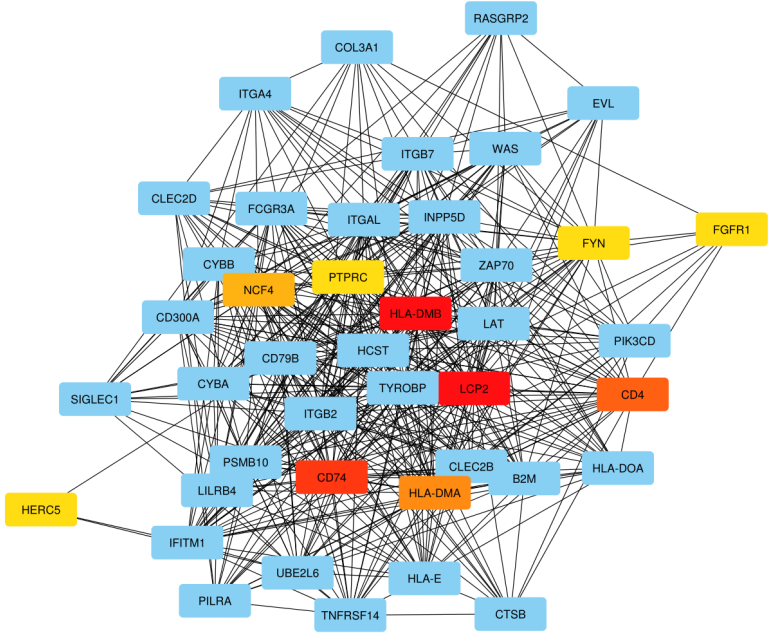

Closeness Measure

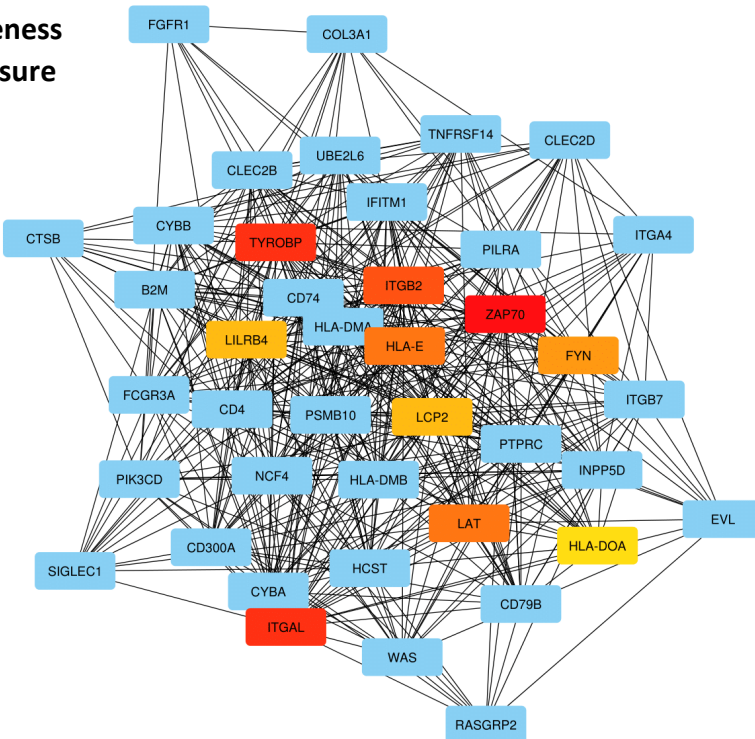

Bottleneck Measure

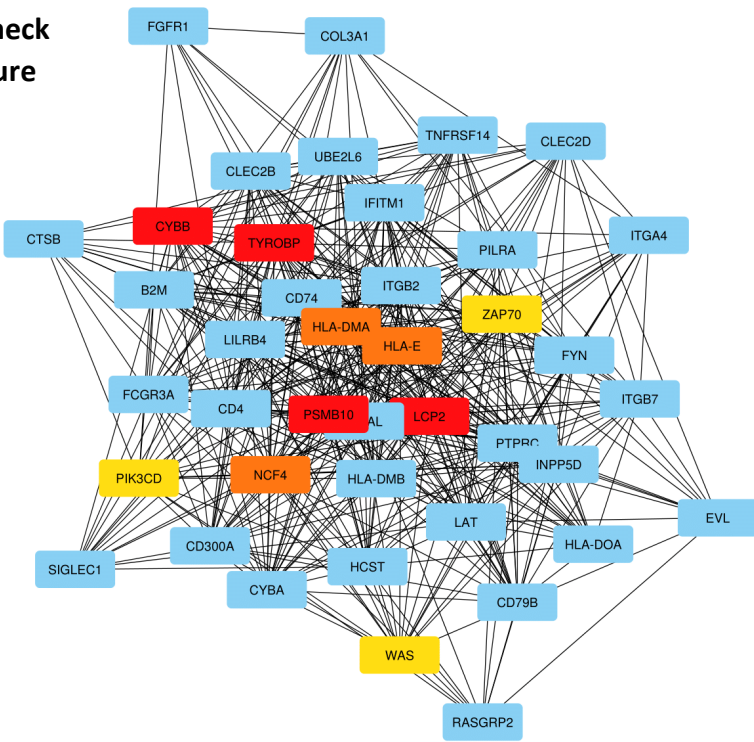

Supplement: Supplementary file 1 [file jpm-11-01129-s001.zip › Figure S3.pdf]
